# A Word to Patients—“How to Treat Your Doctor”

**Published:** 1863-10

**Authors:** 


### A WORD TO PATIENTS—"HOW TO TREAT YOUR DOCTOR."

The question is, what are our country friends to do when they come here for advice. We honestly advise them to do that most difficult of all things, to shut their mouths and their ears. Choose your adviser, and address him as a gentleman; do not try to cross-examine him as though he were a thief, and you another. You are quite incapable of understanding him without all your limited powers of attention. Don't try to be "smart;" don't ask him if he "ever saw or heard of such a case before." If you do he will think you a conceited, selfish fool. Don't ask him to trust you; if he investigates your case faithfully, remember he does it for the fee. If his opinion is worth having, it is worth paying for. He has always quite as many charity patients whom he knows as he can conveniently attend to. Never continue running to his house at daylight, because the poor man is probably asleep, and he will lose interest in your case if you persecute him and his family. When you have had his opinion, and he has answered your questions, go away; his office is not a saloon for entertainment. Never pick your teeth nor clean your nails when talking to him. If you smoke or chew, do neither in his office, if you wish him to consider you a decent man. Finally, if you come here to consult a mesmerist, an herb or Indian doctor, or a hydropathist, or an academy doctor, do it with faith, and do it only; don't distract your miserable brain with the opinions of others. If you are not a reading and thinking man, if you have no Encyclopœdia at home, and must depend upon your own poor judgment or that of a doctor who has no head nor library, and if you believe that the man you are determined to consult can cure you, try him in Heaven's name; swallow his physic, even if he pour eleven kinds into one tumbler; he will not probably kill you on the spot, for that would be very foolish; and these people ar always, as Bacon says of the ant, "wise creatures for themselves." They want you to live as long as possible, so that you may take and pay for at least a hundred bottles, perhaps two, and then when death does come you will die happier, because you at least had your own way, and did not get kicked and cuffed into some one else's path who knew no more than yourself. You can do no more than use the brains God has given you, and we have generally noticed those who have the smallest stock, enjoy the uncontrolled use of them with the greatest zest.—*Scalpel.*

